# Account of a Singular Calculous Degeneration of the Scrotum, for Which the Whole of That Part Was Successfully Removed

**Published:** 1827-12

**Authors:** Valentine Mott

**Affiliations:** Professor of Surgery in Rutger's Medical College, New-York.


REMOVAL OF THE SCROTUM.

*Account of a singular Calculous Degeneration of the Scrotum, for which the whole of that part was successfully removed.* By VALENTINE MOTT, M.D. Professor of Surgery in Rutger's Medical College, New-York. [Extracted from the *Philadelphia Journal.*]

IN the practice of surgery, we frequently observe very singular morbid alterations of texture, which are worthy of being recorded, notwithstanding our inability to account for their production. None of the works that we have examined contain a description of such a degeneration as that we are about to describe, nor have we ever met with another instance of a similar kind. It may, therefore, be useful to state the fact, as a contribution towards a more complete history of the morbid anatomy of the scrotum.

In the summer of 1824, I was requested to visit J. R., aged about seventy-three, a wealthy farmer residing upon Long Island. His health had been declining for two or three years, from an affection of his stomach, accompanied, as he stated, with an uncommon disease of the scrotum. The latter complaint had so far increased within the last year as materially to injure his health, in consequence of an ulceration and very fetid discharge therefrom. The constant and severe burning which he experienced in the region of the pylorus, with an ejection of the contents of the stomach shortly after eating, together with frequent acid eructations and costiveness, led to the fear that there was some organic derangement of the lower orifice of the stomach.

As the disease of the scrotum was the particular object of my visit, I requested permission to examine it. It exhibited a monstrous, and to me a very unique appearance, reaching fully two-thirds the length of his thighs, being from twelve to fifteen times its ordinary bulk, and studded, particularly on each edge, it being flattened anteriorly and posteriorly, with several dozen tumors, of a stony hardness, covered with the integuments, from the size of nutmegs to that of a large pea. It resembled an enormous bunch of grapes, or more closely some morbid conditions of the pancreas and spleen, which we have occasionally met with. The tumors had all a very white appearance, and the integuments of two or three of the largest, having been ulcerated for upwards of a year, poured forth a constant and very fetid discharge. At these openings white bodies were seen, which, when touched with a probe, felt of a stony hardness. A white substance resembling mortar was discharging from these openings, which resulted from the crumbling away of the calculi and the combination of this substance with the fluid from the ulcers.

This state of the scrotum was upwards of twenty years' duration, and had been gradually increasing, the tumors multiplying as the scrotum augmented in size. The patient knew of no cause to which it could be ascribed.

From its size and weight, as well as the loathsome nature of the discharge, he became desirous to have it removed, if practicable and proper. His health being sufficiently good, and the testes appearing to move freely in the diseased mass, led me to recommend that the operation should be performed.

An incision was made around the root or base of the scrotum, beginning on each side of the under part of the penis, at a point a little above the scrotum, so that some integument of this part of the penis in a diseased state was also removed, and carried down to the perineum, leaving an angular portion of the scrotum below, of about an inch in length. Cautiously cutting through the diseased integuments and the subcutaneous cellular structure, the vaginal coat of each testis was readily discovered and avoided. The whole of the morbid mass was removed by cautious dissection, leaving the tunica vaginalis on each side sound and unopened. Numerous arteries were secured during the dissection, in the integuments, as well as several large ones in the septum scroti.

The perineal portion of the scrotum was susceptible of very considerable elongation, but it was altogether insufficient to cover the testes. A new covering for them, therefore, could only be looked for from the granulatory process. Light dressings of lint, compress, and a T bandage, were applied for the first two days, followed by emollient poultices to favor the second mode of healing.

Suppuration and granulation being well established, the new scrotum was increased and fashioned by the use of adhesive straps.

His complete recovery from the operation, and the reproduction of a scrotum, was not interrupted by any circumstance. Three years have now elapsed, and he enjoys excellent health, being occasionally obliged to take, for a week or two, a few grains of the Subnitrate of Bismuth, to remove the affection of his stomach, which, before the operation was performed, threatened to become an organic disease.

